# Association Between Immunotherapy and Generalization in Ocular Myasthenia Gravis as a Function of Antibody Status

**DOI:** 10.31083/RN50142

**Published:** 2026-06-26

**Authors:** Siyu Xie, Ran Guo, Bo Jin, Wenting Guo, Yifan Cheng, Wenli Song, Chunrong Li, Manman Zhang, Yiqi Wang

**Affiliations:** ^1^The Second Clinical Medical College of Hangzhou Normal University, 310014 Hangzhou, Zhejiang, China; ^2^Center for Pain Management Department, Zhejiang Provincial People’s Hospital (Affiliated People’s Hospital, Hangzhou Medical College), 310014 Hangzhou, Zhejiang, China; ^3^Department of Neurology, Sir Run Run Shaw Hospital, School of Medicine, Zhejiang University, 310016 Hangzhou, Zhejiang, China; ^4^Center for Rehabilitation Medicine, Department of Neurology, Zhejiang Provincial People’s Hospital (Affiliated People’s Hospital, Hangzhou Medical College), 310014 Hangzhou, Zhejiang, China

**Keywords:** myasthenia gravis, ocular, myasthenia gravis, generalized, immunotherapy, treatment outcome

## Abstract

**Background:**

Ocular myasthenia gravis (OMG) is an autoimmune disorder characterized by weakness of the extraocular muscles. This study was developed to investigate the relationship between immunotherapy efficacy and OMG generalization as a function of antibody status, and to explore the efficacy of different immunotherapies in preventing OMG generalization.

**Methods:**

This retrospective study included 60 patients admitted to our hospital with initial OMG who were followed for more than two years. Time to generalization was calculated from symptom onset. The proportional hazards assumption was tested using time-dependent covariates, employing Cox regression and Kaplan-Meier analyses to analyze associations for this patient cohort.

**Results:**

Among the 60 patients (28 converted OMG; 32 pure OMG), the median time to generalization was 52 months (interquartile range (IQR): 16–117). Antibody positivity was an independent risk factor for generalization (hazard ratio (HR) = 3.89, 95% confidence interval [CI]: 1.26–12.01, *p* = 0.018). In antibody-positive patients, immunotherapy significantly reduced generalization risk (HR = 0.17, 95% CI: 0.04–0.62, *p* = 0.008).

**Conclusion:**

The time from OMG onset to generalization can be prolonged. While antibody positivity was independently associated with generalization risk in this study cohort, immunotherapy was linked to a time-stable reduction in this risk among antibody-positive patients. These findings provide a preliminary basis for antibody-status-based individualized treatment in OMG.

## 1. Introduction

Myasthenia gravis (MG) is a chronic autoimmune disease that affects the neuromuscular junction and is characterized by fluctuating muscle weakness. Common symptoms include ptosis, diplopia, dysphagia, limb weakness, and respiratory involvement [1,2]. Approximately 50% of MG patients initially present with ocular symptoms, such as ptosis and/or diplopia, a condition referred to as ocular myasthenia gravis (OMG) [3]. About 50%–80% of OMG patients may develop generalized MG (GMG) characterized by generalized skeletal muscle weakness within 2 years of onset [4,5].

In patients with OMG, the European Federation of Neurological Societies/European Neurological Society (EFNS/ENS) guidelines currently recommend pyridostigmine as first-line therapy [6]. Updated international consensus criteria further advise adding immunosuppressants for patients with acetylcholinesterase inhibitor (AChEI)-unresponsive, functionally limiting symptoms [7]. There is a growing body of evidence supporting the value of immunosuppressive therapy to prevent generalization [8]. Bhanushali et al. (2008) [9] reported that corticosteroids may be more effective than AChEI in treating ocular symptoms, while a 2019 systematic review highlighted the potential of early immunotherapy as a means of delaying or preventing generalization [10]. However, findings from observational studies remain inconsistent, with some reporting uncertain benefits of corticosteroids and azathioprine [11].

Recent studies have identified thymic abnormalities and high acetylcholine receptor (AChR) antibody titers as risk factors for generalization [12,13]. Antibody-negative patients or those with normal thymic morphology may have the disease confined to the ocular compartment for extended periods. In a 2018 study, researchers suggested that corticosteroids may only prevent generalization in patients with thymic hyperplasia [13]. AChR antibody seropositivity is considered to coincide with better response to medical treatment [14], but it remains uncertain as to whether immunotherapy is only effective in preventing generalization among high- but not low-risk OMG patients. Given the potential adverse effects of long-term immunosuppression [15], there is a clear clinical need to determine which OMG patients are most likely to benefit from immunotherapy.

The present study was designed to conduct a preliminary exploration of the relationship between therapeutic response to immunosuppression and OMG generalization as a function of patient antibody status (antibody-positive vs. antibody-negative). In addition, the relative efficacy of different immunotherapeutic regimens in preventing OMG generalization was explored to better inform the management of this disease.

## 2. Materials and Methods

### 2.1 Patient Ascertainment and Data Collection

MG Patients with initial ocular symptoms who were admitted to the Department of Neurology, Zhejiang Provincial People’s Hospital, between January 2013 and March 2023 were retrospectively reviewed. All patients had a follow-up duration of at least two years. Patients with “Pure OMG” were defined as those with exclusively ocular symptoms lasting for 2 years or longer, whereas those with “converted OMG” were defined as those who generalized from OMG to GMG over the follow-up period.

Diagnoses were made by experienced neurologists. Antibody-positive OMG was diagnosed based on the presence of two core components: (1) Detection of a specific pathogenic antibody; and (2) Fluctuating ocular manifestations, including ptosis and/or diplopia. Antibody-negative OMG diagnoses were established through a comprehensive approach in which all of the following criteria were fulfilled: (1) Documentation of characteristic fluctuating ocular symptoms; (2) Confirmation of seronegativity via an extensive antibody assay; (3) Electrophysiological or pharmacological evidence of neuromuscular dysfunction, demonstrated by at least one positive test among the ice pack test, repetitive nerve stimulation, or neostigmine test; and (4) Systematic exclusion of other etiologies that may present with isolated ophthalmoparesis. Patients were considered to have converted OMG when their MG symptoms extended beyond the extraocular muscles, manifesting as any objectively verifiable weakness in the bulbar, limb, or axial muscles upon physical examination.

### 2.2 Generalization Rates

The timing of generalization from OMG to GMG was recorded during the follow-up observation period. The primary endpoint was the time from symptom onset to generalization. For the 13 patients already generalized at the first visit, the event time was defined as the interval from symptom onset to the first documented evidence of generalization. Patients who did not develop generalized disease were right-censored at their last follow-up. Based on clinical evolution, patients were classified into the pure OMG group and the converted OMG group.

### 2.3 Immunotherapy and Generalization

Clinical data were collected for each patient during their first visit. Comparative analyses were performed between the pure and converted OMG groups across eight clinical variables: age at onset, gender, onset time, presenting symptom (ptosis vs. diplopia), early or late onset, antibody status, presence of thymoma, and history of thymectomy. Antibody positivity was defined as the presence of any of the following: acetylcholine receptor antibody (AChR-Ab), muscle-specific kinase antibody (MuSK-Ab), or low-density lipoprotein receptor-related protein 4 antibody (LRP4-Ab). Early-onset OMG (EOMG) was defined by onset ≤50 years of age, while late-onset OMG (LOMG) was defined by onset >50 years of age.

Patients were also classified into immunotherapy groups, defined based on treatment regimens initiated according to clinical discretion during follow-up. These included a corticosteroid monotherapy group (CS-Mono group) and a corticosteroid combination with non-steroidal immunosuppressants (NSIS) group (CS-NSIS group). Patients who received neither corticosteroids nor NSIS throughout the follow-up period were classified into the no immunotherapy group (NI group). In the CS-Mono group, prednisone was the corticosteroid administered to patients. In the CS-NSIS group, prednisone was combined with tacrolimus or azathioprine as NSIS agents. The timing of immunotherapy initiation was defined as the interval between symptom onset and the start of immunosuppressive treatment.

Given the distinct characteristics of different patient subgroups in this study, different analytical approaches were applied. In the antibody-positive group, the independent prognostic value of immunotherapy was assessed via multivariable Cox proportional hazards regression analysis. In the antibody-negative subgroup, in contrast, a reliable Cox model could not be fitted due to the small sample size and complete separation in the data. For this group, the analysis was therefore performed using the Kaplan-Meier approach with log-rank testing.

### 2.4 Statistical Analyses

All statistical analyses were performed using R (v4.2.2, R Core Tame, Vienna, Austria) and the MSTATA software (v0.98, www.mstata.com). Continuous variables were expressed as mean ± SD (for normally distributed data) or median with interquartile range (for non-normally distributed data). Categorical variables were presented as frequencies and percentages. Fisher’s exact test was used for categorical variables, while *t*-tests were used for normally distributed continuous variables, and Mann-Whitney U tests were used for continuous variables with skewed distributions. A Cox proportional hazards regression approach was used to identify independent risk factors for generalization, and results were reported as hazard ratios (HRs) with 95% confidence intervals (CIs). The proportional hazards assumption was assessed using time-dependent covariates. A post hoc power analysis based on the observed events and HR estimates indicated approximately 80% power for the primary outcome at a two-sided α of 0.05. Survival probabilities were estimated via Kaplan-Meier analysis, and corresponding survival curves were generated with generalization from OMG to GMG as the endpoint. In the antibody-positive subgroup, Fisher’s exact test was used for pairwise comparisons of generalization proportions across the three treatment groups (NI, CS-Mono, CS-NSIS). A Bonferroni-corrected significance threshold of *p* < 0.0167 was applied to account for multiple comparisons. For all other statistical analyses, a two-sided *p* < 0.05 was considered significant.

## 3. Results

**Fig. 1.**
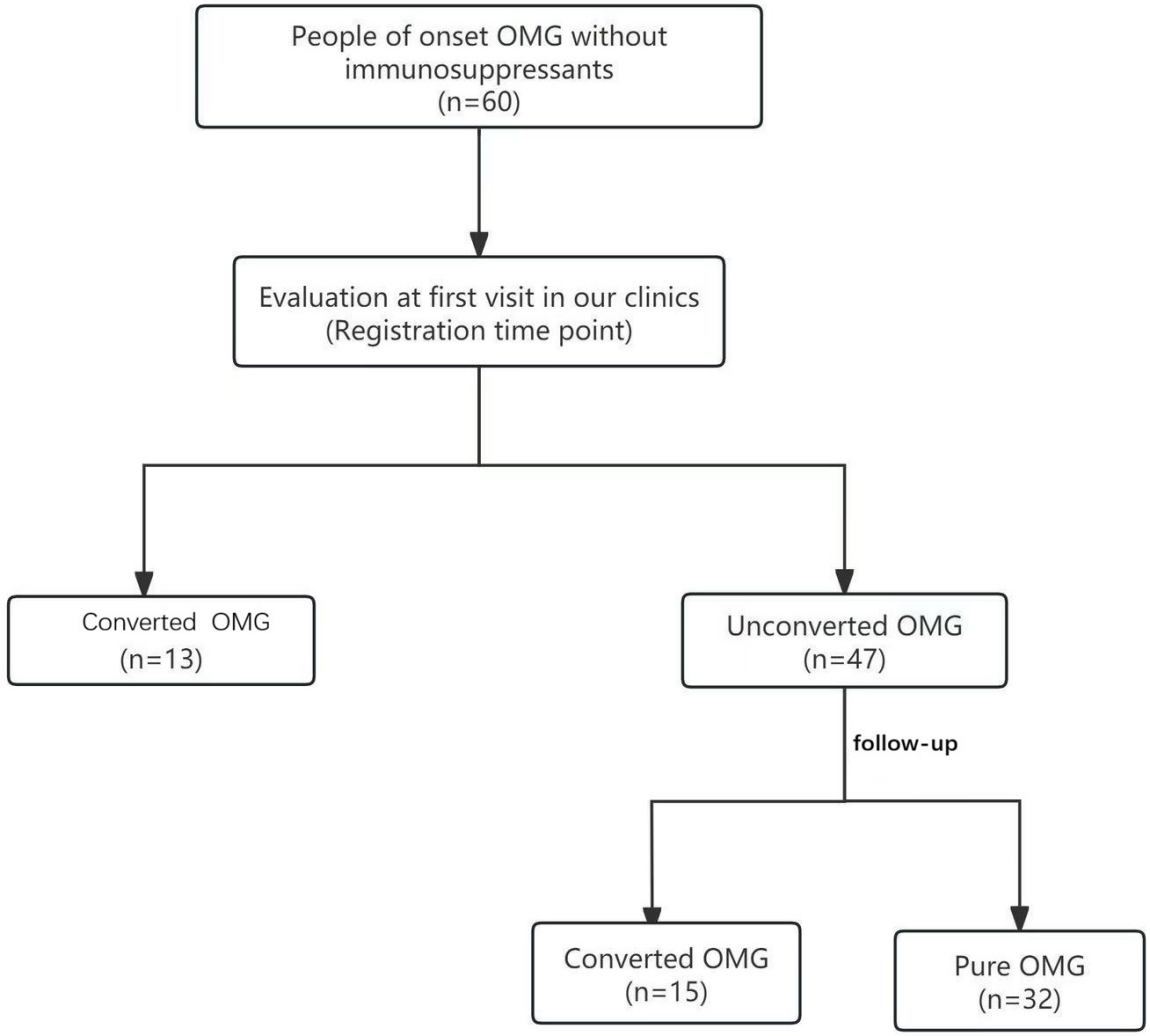
**Flowchart for the selection of included patients**. OMG, ocular myasthenia gravis.

A total of 60 patients were included in this study, and complete follow-up data were available for all participants. At their first visit, 13 patients had already generalized from OMG to GMG. During the follow-up observation of the remaining 47 patients, another 15 converted OMG patients were recorded (Fig. 1). The mean age at the onset of OMG was 38.85 ± 20.99 years, and the overall male-to-female ratio was approximately 1:1.7. The cohort included 37 EOMG patients and 23 LOMG patients. Antibody positivity was identified in 39 patients (65.0%), while 21 patients (35.0%) were seronegative. With respect to initial symptoms, 47 patients (78.3%) presented with isolated ptosis, while 13 (21.7%) presented with diplopia, with or without ptosis. Thymoma was detected in 8 patients (13.3%), and thymectomy was performed in 17 patients (29.3%). The rate of antibody positivity was significantly higher in the converted OMG group as compared to the pure OMG group (85.7% vs. 46.9%, *p* = 0.002) (Table 1). No other clinically meaningful differences in baseline characteristics were observed between these groups.

**Table 1.** **Clinical characteristics of 60 MG patients who initially presented with purely ocular symptoms**.

Clinical characteristic		Total	Pure OMG	Converted OMG	*p*-value
		(n = 60)^1^	(n = 32)^1^	(n = 28)^1^	
Gender (%)					0.694^2^
	Male	22 (36.7%)	11 (34.4%)	11 (39.3%)	
	Female	38 (63.3%)	21 (65.6%)	17 (60.7%)	
Disease duration, months (IQR)		60 (36,132)	70 (46,157)	52 (16,117)	0.071^3^
Onset age, years (Mean ± SD)		38.85 ± 20.99	41.22 ± 21.65	36.14 ± 20.26	0.352^4^
Onset symptom (%)					0.065^2^
	No diplopia	47 (78.3%)	28 (87.5%)	19 (67.9%)	
	Diplopia	13 (21.7%)	4 (12.5%)	9 (32.1%)	
Onset (%)					0.696^2^
	Early onset	37 (61.7%)	19 (59.4%)	18 (64.3%)	
	Late onset	23 (38.3%)	13 (40.6%)	10 (35.7%)	
Thymoma (%)					>0.999^5^
	No	52 (86.7%)	28 (87.5%)	24 (85.7%)	
	Yes	8 (13.3%)	4 (12.5%)	4 (14.3%)	
Thymectomy (%)					0.424^2^
	No	41 (70.7%)	24 (75.0%)	17 (65.4%)	
	Yes	17 (29.3%)	8 (25.0%)	9 (34.6%)	
Antibody (%)					0.002^2^ ^**^
	Negative	21 (35.0%)	17 (53.1%)	4 (14.3%)	
	Positive	39 (65.0%)	15 (46.9%)	24 (85.7%)	

Note: Early-onset age ≤50 years; Late-onset age >50 years; Thymectomy status was unknown for 2 patients (both in the converted OMG group). Percentages for Thymectomy are calculated based on the 58 patients with known data. ^1^n (%); Median (IQR); Mean ± SD **Statistically significant, *p* < 0.01. ^2^Pearson’s Chi-squared test. ^3^Wilcoxon rank sum test. ^4^Welch Two-Sample *t*-test. ^5^Fisher’s exact test. Abbreviations: MG, myasthenia gravis; IQR, interquartile range; SD, standard deviation.

**Fig. 2.**
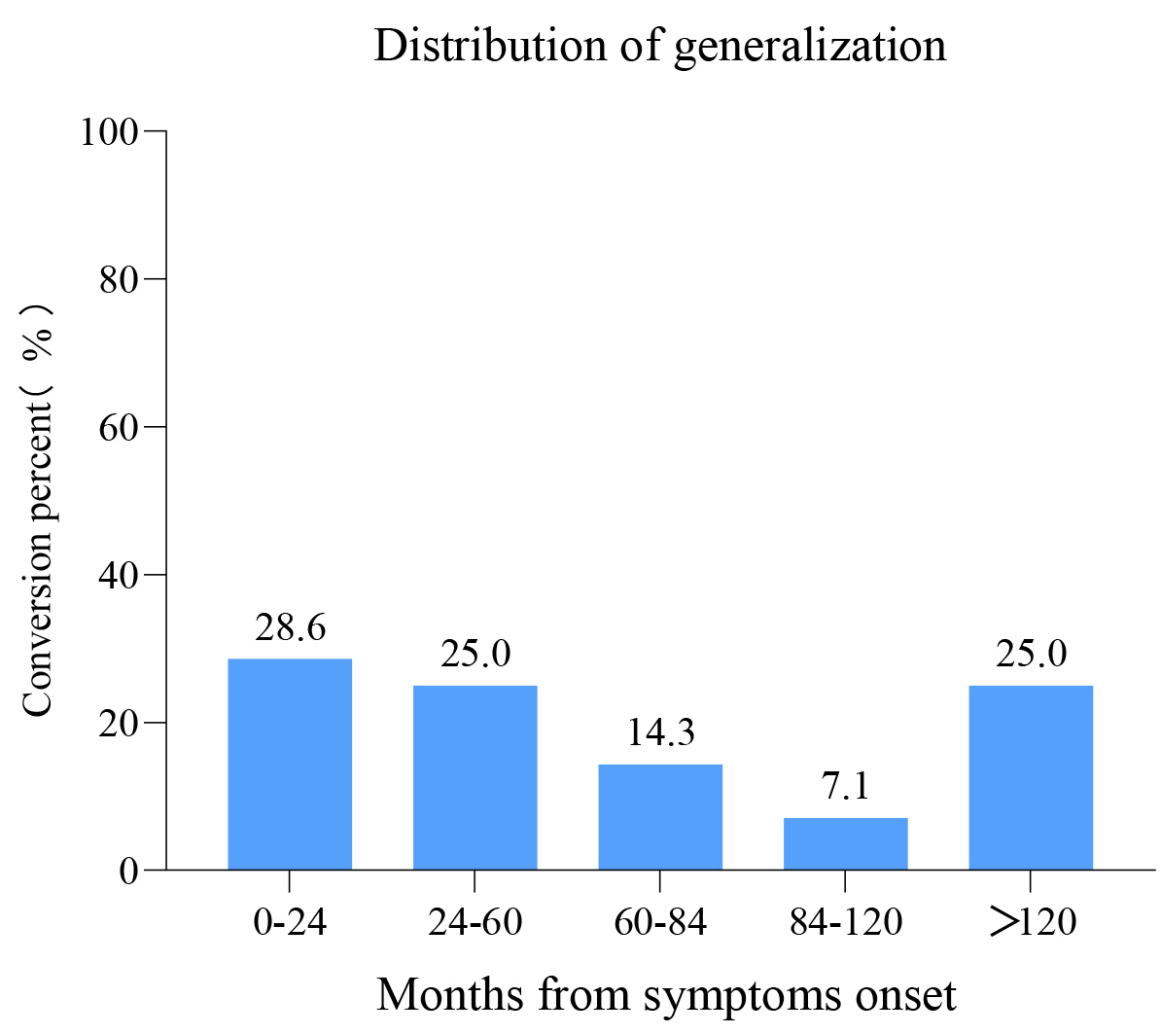
**The distribution of time to generalization in 28 patients with OMG who converted to GMG**. GMG, generalized MG.

Among the 28 patients who converted to GMG, 28.6% generalized within two years of symptom onset, and 53.6% within five years. Notably, 25.0% of generalizations occurred more than 10 years after disease onset (Fig. 2). Among the 28 converted OMG patients, the median time to generalization was 52 months (interquartile range [IQR]: 16–117), and the mean age at generalization was 36.14 ± 20.26 years (Table 1). Patients were categorized into CS-Mono, CS-NSIS, and NI groups based on immunotherapy regimens. The distribution of specific immunosuppressive agents and the timing of treatment initiation are summarized in Table 2.

**Table 2.** **Distribution of immunosuppressive regimens and timing of treatment initiation across treatment groups**.

Treatment group	Specific immunosuppressive regimen	n (%)	Time from disease onset to initiation of current regimen (months, IQR)
NI group	No immunotherapy	36 (60.0%)	**-**
CS-Mono group	Prednisone	9 (15.0%)	1.97 (0.00–8.40)
CS-NSIS group	Prednisone, Azathioprine	6 (10.0%)	19.50 (2.36–186.50)
	Prednisone, Tacrolimus	9 (15.0%)	22.03 (3.77–200.90)

Note: Patients were classified according to their overall immunotherapy regimen during follow-up, and specific agents were identified from medical records. Treatment timing was defined as the interval between disease onset and initiation of immunotherapy. Complete dosing information was not available in all cases; commonly documented regimens included azathioprine 50 mg twice daily and tacrolimus 1.5 mg twice daily. Abbreviations: NI, no immunotherapy; CS-Mono, corticosteroid monotherapy; NSIS, non-steroidal immunosuppressants.

Multivariable Cox proportional hazards analysis was performed on six clinical parameters, pre-selected based on their clinical importance, to identify independent risk factors for generalization (Table 3). The model confirmed the absence of severe multicollinearity (all VIFs <5). Antibody positivity emerged as a significant risk factor for conversion to GMG (HR = 3.89, 95% CI: 1.26–12.01, *p* = 0.018). Post hoc power analysis indicated that the study had approximately 80% power to detect the primary association.

**Table 3.** **Cox proportional hazards regression analysis of risk factors for generalization in the overall 60-patient cohort**.

Variables	Multivariate analysis	*p*-value
	HR (95% CI)	
Gender, female vs male	1.01 (0.43–2.34)	0.990
Onset, early onset vs late onset	1.57 (0.62–3.97)	0.345
Diplopia, yes vs no	1.40 (0.50–3.98)	0.523
Antibody, positive vs negative	3.89 (1.26–12.01)	0.018^*^
Thymoma, yes vs no	1.22 (0.31–4.82)	0.774
Thymectomy, yes vs no	0.65 (0.19–2.15)	0.476

Note: Early-onset age ≤50 years; Late-onset age >50 years. Abbreviations: HR, hazard ratio; CI, confidence interval. *Statistically significant, *p* < 0.05.

Kaplan-Meier curves were used to assess the relationship between immunotherapy treatment and rates of OMG generalization. In antibody-positive patients (Fig. 3a), immunotherapy significantly reduced the risk of generalization from OMG to GMG (Log-rank *p* = 0.043). However, in antibody-negative patients (Fig. 3b), the survival curves between the immunotherapy and NI groups exhibited a complex time-dependent relationship, with early crossover during follow-up. Neither the Log-rank test (*p* = 0.675) nor the Breslow test (*p* = 0.757) indicated any significant association between immunotherapy and generalization in these antibody-negative patients.

**Fig. 3a.**
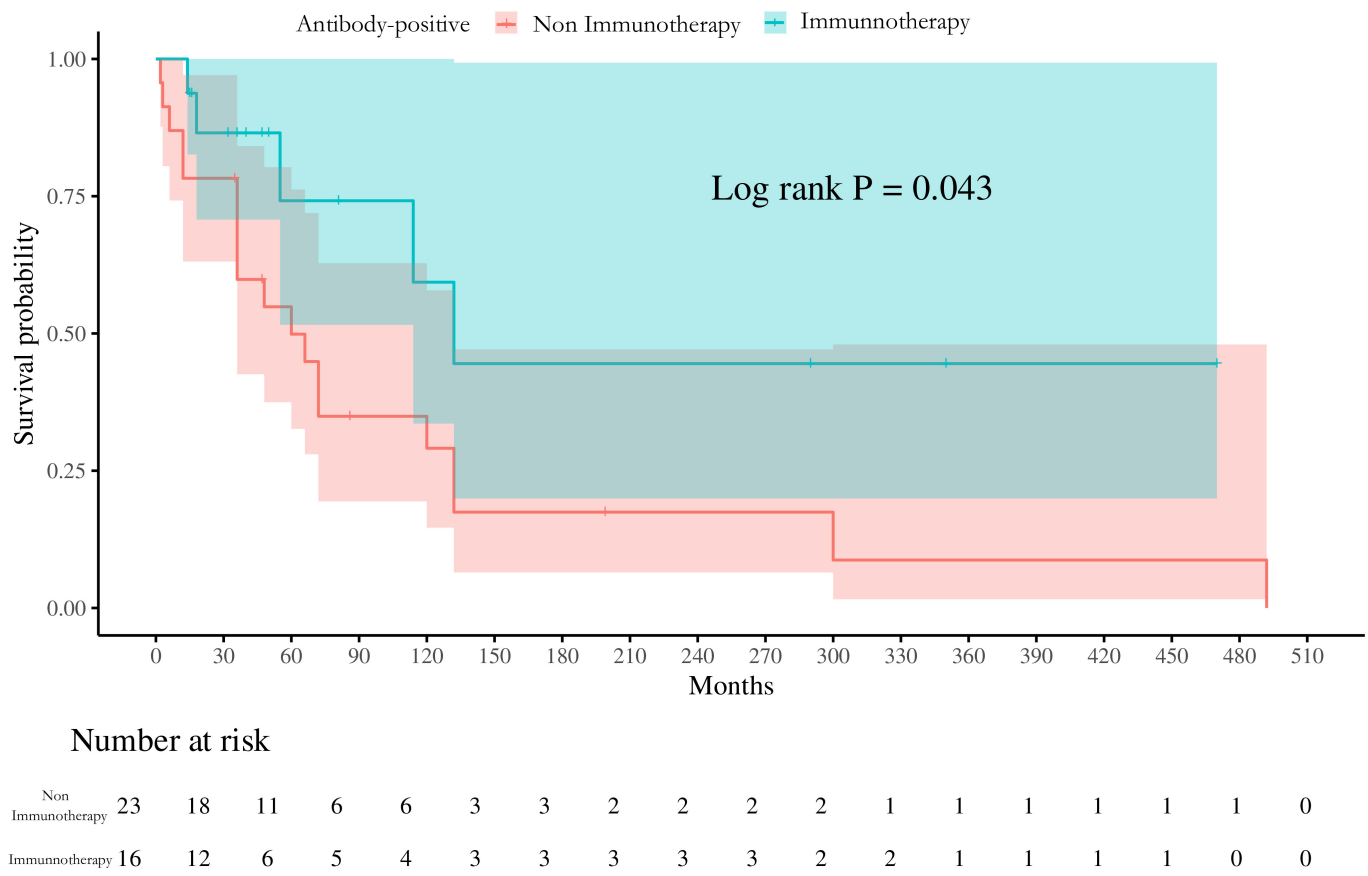
**Kaplan-Meier curves for survival without generalization according to immunotherapy treatment status among antibody-positive patients**.

**Fig. 3b.**
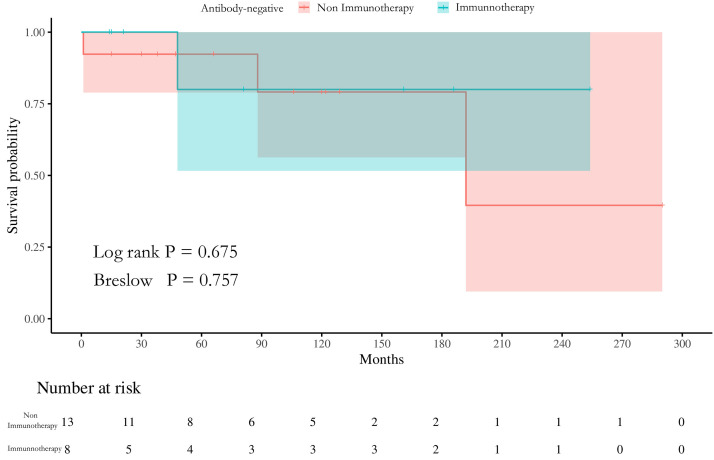
**Kaplan-Meier curves for survival without generalization according to immunotherapy treatment status among antibody-negative patients**.

To further verify immunotherapy as an independent protective factor in antibody-positive patients, we performed a multivariate Cox regression analysis (Table 4). After adjusting for potential confounding factors, including sex, thymoma, age at onset, and initial symptoms, immunotherapy associated with a reduced risk of generalization (HR = 0.17, 95% CI: 0.04–0.62, *p* = 0.008). The proportional hazards assumption was satisfied for immunotherapy in this model (*p* = 0.299; **Supplementary Table 1**). Thymectomy was also associated with a nominally reduced risk of generalization (HR = 0.23, 95% CI: 0.05–0.96, *p* = 0.044). However, the proportional hazards assumption was violated for this variable (*p* = 0.006, **Supplementary Table 1**), and the association was not confirmed in time-stratified sensitivity analyses. As such, this finding should be interpreted with caution. Due to the inherent data limitations for the antibody-negative subgroup, a multivariate Cox regression analysis was not performed for these patients. The survival analysis for this subgroup is presented solely in the form of the survival curve (Fig. 3b), which revealed no significant benefit from immunotherapy with respect to the risk of generalization.

**Table 4.** **Cox proportional hazards regression analysis of risk factors for generalization among antibody-positive patients**.

Variables	Univariate analysis		Multivariate analysis	
	HR (95% CI)	*p*-value	HR (95% CI)	*p*-value
Gender, female vs male	1.21 (0.51–2.86)	0.670	3.17 (0.89–11.24)	0.074
Onset, early onset vs late onset	1.32 (0.56–3.11)	0.523	1.02 (0.37–2.86)	0.964
Diplopia, yes vs no	1.32 (0.52–3.39)	0.560	0.60 (0.18–2.07)	0.423
Thymoma, yes vs no	0.84 (0.28–2.49)	0.748	1.12 (0.29–4.36)	0.870
Thymectomy, yes vs no	0.66 (0.28–1.57)	0.348	0.23 (0.05–0.96)	0.044^*^
Immunotherapy, yes vs no	0.38 (0.14–1.04)	0.059	0.17 (0.04–0.62)	0.008^**^

Notes: Early-onset age ≤50 years; Late-onset age >50 years; The proportional hazards assumption was satisfied for immunotherapy (*p* = 0.299) but violated for thymectomy (*p* = 0.006). Abbreviations: *Statistically significant, *p* < 0.05, **Statistically significant, *p* < 0.01.

**Fig. 4.**
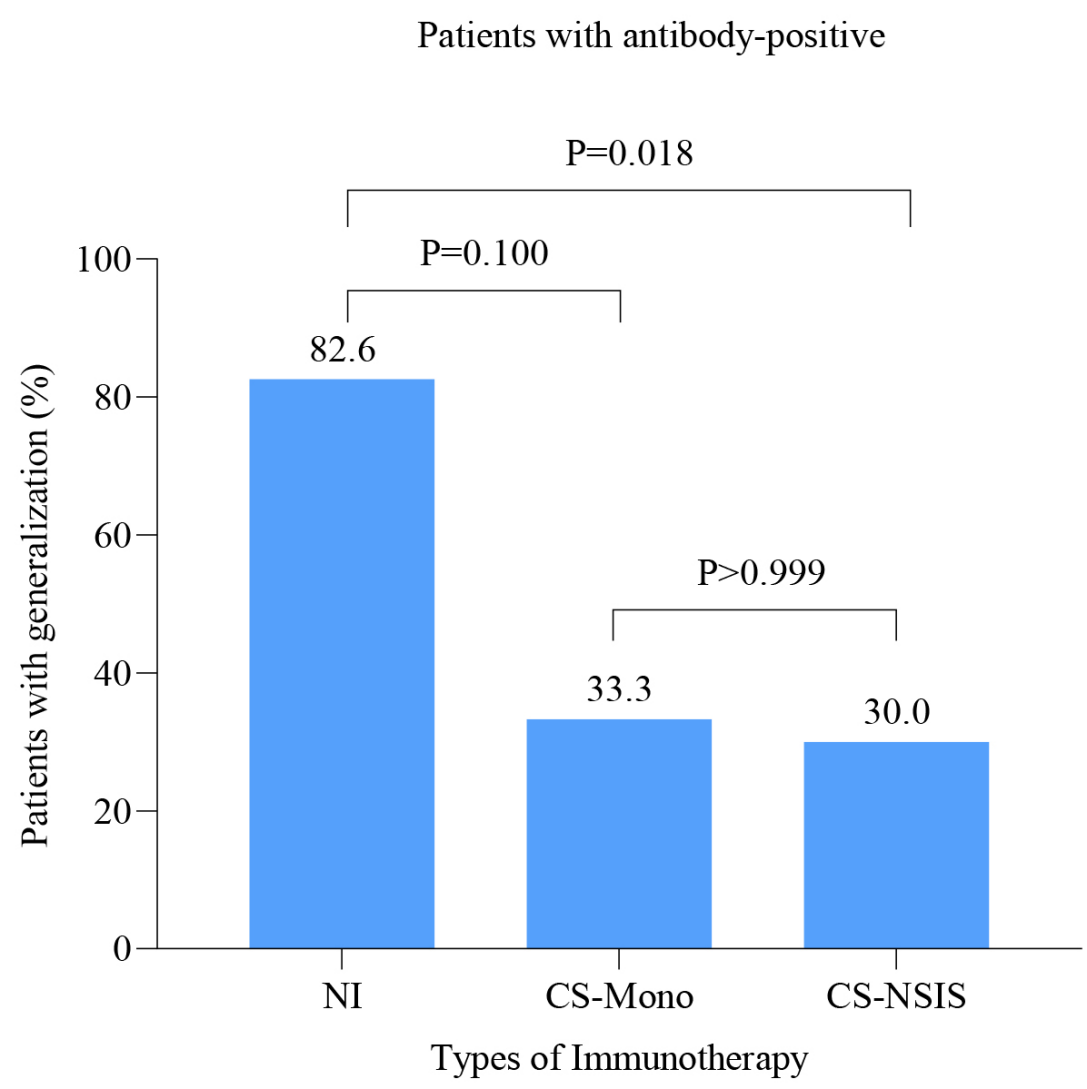
**Generalization rates for antibody-positive OMG patients (n = 39) classified according to immunotherapy regimens. **Note: *p* values are Bonferroni-corrected for three comparisons. No comparisons remained statistically significant at α = 0.0167.

In the antibody-positive subgroup (n = 39), generalization rates were 82.6% (19/23) in the NI group, 33.3% (2/6) in the CS-Mono group, and 30.0% (3/10) in the CS-NSIS group. Pairwise Fisher’s exact test analyses with Bonferroni correction revealed no statistically significant differences among the three groups. However, numerical trends suggested a lower generalization rate in the CS-NSIS group compared to the NI group (30.0% vs. 82.6%, corrected *p* = 0.018). The rate for the CS-Mono group did not differ significantly from the NI group (corrected *p* = 0.100), nor was there any significant difference between the CS-Mono and CS-NSIS groups (corrected *p* > 0.999) (Fig. 4, **Supplementary-STROBE_checklist**).

## 4. Discussion

In this study, the overall generalization rate for OMG patients was 46.7%, with 25.0% of generalization occurring more than 10 years after initial symptom onset. Antibody positivity was identified as an independent risk factor for generalization. Among antibody-positive OMG patients, immunotherapy was associated with a reduced risk of generalization, whereas it showed no association with generalization among antibody-negative OMG patients. 

Clear ethnicity-related differences in the incidence, phenotypic presentation, and generalization rate of OMG have been reported in prior studies [16,17]. The low 2-year generalization rate in our cohort (13.3%) aligns with findings from other Asian populations, such as the 7.7% rate reported by Teo et al. in Singapore [18] and the 12.4% progression rate beyond 2 years among Chinese children reported by Gui et al. [19]. Several factors may explain these relatively low rates. Childhood-onset OMG is more common in Asian populations, with OMG being four times more frequent than in European children, and progression to generalized disease being less common. Immunogenetic differences, including Human Leukocyte Antigen Class I Molecule B-46 Alpha Chain (HLA-B*46) and Human Leukocyte Antigen Class II DRB1-9 Beta Chain (DRB1*09) in Asian children versus Human Leukocyte Antigen Class II DRB1-4 Beta Chain (HLA-DRB1*04) in European children with generalized disease, may contribute to this discrepancy [20]. Southern Chinese populations also show a higher proportion of seronegative MG, often with fewer thymomas and predominantly ocular presentation, which may be linked to a lower risk of generalization [21]. Notably, 30.0% of generalization events in our cohort occurred beyond 10 years, consistent with a 10-year risk of 37.8% reported previously [22], highlighting the need for long-term surveillance.

Consistent with previous studies focused on generalization-related risk factors [22,23,24], our results also confirm that antibody positivity is a strong predictor of generalization. Other studies have also identified thymoma, positive repetitive nerve stimulation (RNS) findings, and older age at onset as predictive factors [2,15]. We did not observe a significant association between thymoma and generalization in our cohort, possibly due to the relatively small number of thymoma cases.

While previous research has suggested a potential benefit of immunotherapy in reducing the risk of generalization [23,24], robust prospective randomized controlled trials remain limited [8]. In 2007, a case against prednisone therapy and thymectomy for OMG patients was presented [25]. A 2018 study from Germany did not find corticosteroids to be protective [13]. In 2009, Kupersmith [26] reported that prednisone delayed the onset of GMG and controlled diplopia symptoms. Our findings suggest that immunotherapy helps prevent generalization. In addition to immunotherapy, the association between thymectomy and reduced generalization risk in antibody-positive patients was nominally significant but did not satisfy the proportional hazards assumption, indicating an unstable effect. Coupled with unmeasured factors such as surgical approach, thymic pathology, timing, and postoperative management, its prophylactic role in OMG remains uncertain [27]. Female antibody-positive patients also exhibited a trend toward a higher risk of generalization. Although not statistically significant, this may be clinically relevant, as female sex has been reported as an independent predictor of generalization with earlier progression [28]. Female antibody-positive patients may therefore warrant closer follow-up.

Importantly, few studies have stratified OMG patients by antibody status when assessing immunotherapy outcomes. A 2024 study suggested that immunotherapy may provide greater benefit in antibody-positive patients, a finding consistent with our data [14]. However, that study did not examine specific regimens. Other study has similarly reported reduced generalization risk with immunotherapy in anti-AChR antibody-positive patients [29]. Notably, 20–50% of OMG patients never generalize, and current evidence does not support immunotherapy as a means of preventing generalization in antibody-negative patients. Our study suggests no significant association between immunotherapy and generalization among antibody-negative patients, which is consistent with the notion that antibody-negative OMG may represent a distinct clinical entity with a less prominent immune basis [30]. This indicates that not all patients with OMG require immunotherapy, and long-term use of both corticosteroids and NSIS carries a risk of significant adverse effects [31]. This may suggest that routine long-term immunosuppression solely to prevent generalization should be applied more selectively in this subgroup. Patients with mild or stable symptoms may be managed conservatively, whereas those with disabling or refractory ocular symptoms may benefit from low-dose corticosteroids, with non-steroidal immunosuppressants added when clinically necessary.

Generalization of OMG is thought to result from persistent immune activation and progression to systemic disease. While corticosteroids provide rapid suppression, the addition of azathioprine or tacrolimus may sustain modulation of pathogenic immune responses and improve disease stability during steroid tapering, potentially reducing generalization risk [32,33,34]. NSIS is more commonly used in GMG (30.0%) than OMG (15.0%), reflecting prioritization in more severe cases [31]. Factors associated with NSIS use include obesity, disease duration, early achievement of minimal manifestation status (MMS), and relapse history [35]. Limited evidence exists to guide immunotherapy decisions specifically in OMG. Further studies are needed to determine whether combination regimens offer additional benefit over monotherapy in preventing generalization.

This study has several limitations. First, the overall sample size was modest, particularly in subgroup analyses, potentially limiting the detection of smaller effects. Nevertheless, a post hoc power analysis indicated approximately 80% power for detecting the primary association between antibody positivity and generalization, supporting the robustness of our main findings. Second, some clinically relevant variables, including exact immunosuppressive dosages, were not systematically collected, potentially introducing residual confounding. Third, multivariable Cox regression could not be performed in the antibody-negative subgroup due to limited sample size, which restricts adjustment for potential confounders in this population.

Given the potential adverse effects of long-term immunosuppression, these findings support individualized treatment decisions based on antibody status and risk profile, though larger prospective studies are needed to confirm these observations.

## 5. Conclusion

In conclusion, among patients initially presenting with purely ocular MG symptoms, 46.7% eventually developed GMG. Antibody positivity was identified as a robust risk factor for generalization. Immunotherapy significantly reduced the risk of generalization in antibody-positive patients, with a time-stable protective effect, but no significant association was observed in antibody-negative patients. These findings provide a preliminary basis for individualized, antibody-status-guided treatment decisions in OMG.

## Data Availability

The datasets generated during the current study are available from the corresponding author on reasonable request

## Abbreviations

AChEI, acetylcholinesterase inhibitor; AChR, acetylcholine receptor; AChR-Ab, acetylcholine receptor antibody; CS-Mono, corticosteroid monotherapy; CS-NSIS, corticosteroids combination with non-steroidal immunosuppressants; EOMG, early-onset myasthenia gravis; EFNS/ENS, European Federation of Neurological Societies/European Neurological Society; GMG, generalized myasthenia gravis; LRP4-Ab, low-density lipoprotein receptor-related protein 4 antibody; LOMG, late-onset ocular myasthenia gravis; MG**, **myasthenia gravis; MuSK-Ab, muscle-specific kinase antibody; MMS, minimal manifestation status; NSIS, non-steroidal immunosuppressants; OMG, ocular myasthenia gravis; PH, proportional hazards.
