# Supplementary material for: Association Between Immunotherapy and Generalization in Ocular Myasthenia Gravis as a Function of Antibody Status
Source: Rev Neurol. 2026 Jun 26;81(6):50142. doi: 10.31083/RN50142 (PMC13339795; doi:10.31083/RN50142)
Supplement: Supplementary file 1 [file 1576-6578-81-6-50142-s1.zip › Supplementary Material.docx]

**Supplementary Table 1. Proportional hazards assumption test for core variables**

| **Model** | Variable | Time-interaction P |
| --- | --- | --- |
| Whole cohort | Antibody positive | **0.095** |
|  | Immunotherapy | **0.550** |
|  | Thymectomy | **0.554** |
| Antibody‑positive subgroup | Immunotherapy | **0.299** |
|  | Thymectomy | **0.006** |

**Note:** PH assumption tested via time‑dependent covariate analysis. P > 0.05 indicates that the assumption was satisfied.
